# Electronic audit and feedback intervention with action implementation toolbox to improve pain management in intensive care: protocol for a laboratory experiment and cluster randomised trial

**DOI:** 10.1186/s13012-017-0594-8

**Published:** 2017-05-25

**Authors:** Wouter T. Gude, Marie-José Roos-Blom, Sabine N. van der Veer, Evert de Jonge, Niels Peek, Dave A. Dongelmans, Nicolette F. de Keizer

**Affiliations:** 1 Department of Medical Informatics, Academic Medical Center, Amsterdam Public Health research institute, University of Amsterdam, Amsterdam, The Netherlands; 2 National Intensive Care Evaluation (NICE) foundation, Amsterdam, The Netherlands; 3 MRC Health eResearch Centre, Division of Informatics, Imaging and Data Sciences, Manchester Academic Health Science Centre, The University of Manchester, Manchester, UK; 4 Department of Intensive Care Medicine, Leiden University Medical Center, Leiden, The Netherlands; 5 NIHR Greater Manchester Primary Care Patient Safety Translational Research Centre, Manchester Academic Health Science Centre, The University of Manchester, Manchester, UK; 6 Department of Intensive Care Medicine, Academic Medical Center, University of Amsterdam, Amsterdam, The Netherlands

**Keywords:** Intensive care, Medical audit, Feedback, Quality improvement, Quality indicators, Randomised controlled trial

## Abstract

**Background:**

Audit and feedback is often used as a strategy to improve quality of care, however, its effects are variable and often marginal. In order to learn how to design and deliver effective feedback, we need to understand their mechanisms of action. This theory-informed study will investigate how electronic audit and feedback affects improvement intentions (i.e. information–intention gap), and whether an action implementation toolbox with suggested actions and materials helps translating those intentions into action (i.e. intention–behaviour gap). The study will be executed in Dutch intensive care units (ICUs) and will be focused on pain management.

**Methods and design:**

We will conduct a laboratory experiment with individual ICU professionals to assess the impact of feedback on their intentions to improve practice. Next, we will conduct a cluster randomised controlled trial with ICUs allocated to feedback without or feedback with action implementation toolbox group. Participants will not be told explicitly what aspect of the intervention is randomised; they will only be aware that there are two variations of providing feedback. ICUs are eligible for participation if they submit indicator data to the Dutch National Intensive Care Evaluation (NICE) quality registry and agree to allocate a quality improvement team that spends 4 h per month on the intervention. All participating ICUs will receive access to an online quality dashboard that provides two functionalities: gaining insight into clinical performance on pain management indicators and developing action plans. ICUs with access to the toolbox can develop their action plans guided by a list of potential barriers in the care process, associated suggested actions, and supporting materials to facilitate implementation of the actions. The primary outcome measure for the laboratory experiment is the proportion of improvement intentions set by participants that are consistent with recommendations based on peer comparisons; for the randomised trial it is the proportion of patient shifts during which pain has been adequately managed. We will also conduct a process evaluation to understand how the intervention is implemented and used in clinical practice, and how implementation and use affect the intervention’s impact.

**Discussion:**

The results of this study will inform care providers and managers in ICU and other clinical settings how to use indicator-based performance feedback in conjunction with an action implementation toolbox to improve quality of care. Within the ICU context, this study will produce concrete and directly applicable knowledge with respect to what is or is not effective for improving pain management, and under which circumstances. The results will further guide future research that aims to understand the mechanisms behind audit and feedback and contribute to identifying the active ingredients of successful interventions.

**Trial registration:**

ClinicalTrials.gov NCT02922101. Registered 26 September 2016.

**Electronic supplementary material:**

The online version of this article (doi:10.1186/s13012-017-0594-8) contains supplementary material, which is available to authorized users.

## Background

Yearly, approximately 90,000 critically ill patients are admitted to Dutch intensive care units (ICU) [1]. During their time in the ICU many patients are exposed to adverse experiences; acute pain being a leading stressor [2]. Physical and psychological stresses caused by pain have been associated with increased length of stay, morbidity and poor mental health outcomes [3–5], and affect quality of life even after ICU discharge [6, 7]. Interview studies revealed that almost half of ICU patients experience moderate to severe pain both at rest as well as during procedures [8–11]. There remains a large gap between ideal and actual care with respect to pain management in intensive care, making it a suitable target for quality improvement (QI) strategies, such as audit and feedback (A&F) [12–15]. A&F has been defined as a “summary of clinical performance in a specific area with or without recommendations for action” [16] and aims to support physicians in accurate self-assessment [17].

A Cochrane review of 140 A&F studies [18] concluded that feedback is effective, but with only a median 4.3% absolute improvement (interquartile range 0.5 to 16%). In fact, a quarter of the studies showed negative or no effect. No effect was also found in a previous A&F trial undertaken by the National Intensive Care Evaluation (NICE) quality registry in Dutch intensive care units [19, 20]. A qualitative evaluation explained that physicians experienced several barriers to achieving QI, such as lack of normative standards and benchmarks, inadequate case-mix adjustment, lack of knowledge on how to improve and insufficient allocated time and staff [21]. In attempt to delineate how to most effectively design and deliver A&F interventions, meta-analyses have indicated that A&F may be more effective when baseline performance is low, the source is a supervisor or colleague, it is provided more than once, it is delivered in both verbal and written formats, and when it includes correct solution information, explicit targets, and an action plan [18, 22, 23]. However, there is little information to guide operationalisation of these factors [24], limiting the progress with which we learn how to design and deliver effective A&F interventions [25]. A recent systematic review of electronic A&F trials alone similarly stressed the scarcity of evidence of effectiveness and the underuse of theory [26]. Researchers have consequently called for theory-informed design and evaluation of A&F interventions, and two-armed trials of different approaches to providing A&F to stimulate this progress [27].

### Theoretical framework

**Fig. 1:**
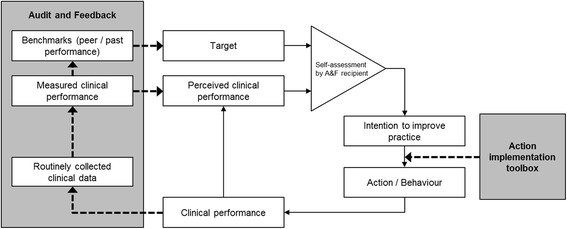
Illustration of hypothesised role played by A&F to improve self-assessments of clinical performance and thus improvement intentions, and the action implementation toolbox to promote behaviour change. Adapted from Carver & Scheier’s Control Theory

Figure 1 depicts our theoretical framework which we based on control theory, specified to reflect the mechanisms through which physicians aim to improve their clinical performance. Control Theory predicts that, if they make a negative assessment of their clinical performance by comparing their performance to a target, physicians develop intentions to take improvement actions and continue these actions until their performance matches the target [28]. However, if they observe discrepancy that is too great, or lack the skills or knowledge on how to improve, recipients may disregard the discrepancy or lower their target to make it more achievable [23, 28, 29].

#### Studying the information–intention gap in audit and feedback

The key assumption behind the success of A&F is that it may improve the accuracy with which physicians self-assess [17]. A&F introduces an external source of information consisting of reports of physicians’ own performance and normative data for comparison measured directly from patient records. To that end, A&F attempts to correct potentially inaccurate perceptions of physicians’ own clinical performance and which targets reflect an appropriate performance level. Inaccurate self-assessments may falsely convince physicians that improvement is or is not desirable, resulting in a “misplaced” focus of improvement intentions [30]. However, this key assumption, i.e. whether A&F yields more accurate self-assessments and adequate improvement intentions, has not been evaluated empirically. This step, to which we will refer as the information–intention gap, is essential in order to initiate behaviour change to improve practice.

#### Closing the intention–behaviour gap in audit and feedback with an action implementation toolbox

In practice, physicians often do not have the time, capacity or skills to interpret feedback and formulate what improvement action is necessary [21, 31, 32]. Similarly, they can experience barriers preventing them to implement their intended actions [33]. Recognizing that providing feedback information alone may be insufficient for recipients to translate their improvement intentions into behaviour, A&F interventions have been frequently combined with successful but both intensive and expensive co-interventions, such as educational outreach visits [34]. These co-interventions presumably work because they convince and help participants to take action to improve patient outcomes. Therefore, an intervention aimed to close the intention–behaviour gap could be very effective. In this study, we hypothesise that augmenting A&F with an action implementation toolbox containing a list of potential barriers in the care process and suggested actions, and supporting materials to facilitate the implementation of actions, helps ICU professionals to turn their intention into action and enhances the likelihood that actions will be completed. Figure 1 illustrates the potential role of the toolbox on effectiveness of A&F.

### Study objectives and hypotheses

The study has two primary objectives around a newly developed electronic A&F intervention that aims to improve clinical performance on recently developed quality indicators relating to pain management in Dutch ICUs:

1. To investigate the extent to which A&F influences physicians’ self-assessments of their performance and intentions to improve practice; and
2. To assess the effects of our electronic A&F intervention with an action implementation toolbox compared to the intervention without toolbox.

We hypothesise that ICUs receiving the A&F intervention will achieve improvements regardless of whether they have access to the toolbox, but that ICUs using the toolbox will achieve larger and faster improvements than those ICUs that do not. Our secondary objective is to understand how the intervention is implemented and used in clinical practice, and how implementation and use affect the intervention’s impact.

## Methods

### Study design

To achieve our objectives, we will perform a mixed-method study consisting of two parts. In part 1, we will undertake a laboratory experiment among individual ICU professionals to assess the impact of A&F on their self-assessments and intentions to improve practice (primary objective 1). Although the experiment will not inform the final A&F intervention design being evaluated in the subsequent field study (i.e. part 2), it might contribute to explaining the A&F effectiveness observed.

In part 2, we will execute a pragmatic two-armed cluster randomised controlled trial (RCT) to determine the impact of the action implementation toolbox on A&F effectiveness (primary objective 2). ICU teams in the intervention group will receive online feedback with action planning functionality including access to an integrated toolbox to facilitate planning and implementing actions. The teams in the control group will receive the same intervention but without access to the toolbox; we provided a more detailed description of the intervention below. Cluster randomisation was chosen because the intervention is implemented at the level of ICUs rather than individual professionals [35]. The lack of a control group receiving no feedback at all was chosen to avoid attrition (because participants expect something in return for contributing data) and statistical power [36, 37]. The study has been designed and will be reported in accordance with the CONSORT statement [38] and the appropriate extensions [39, 40]. The study is registered with ClinicalTrials.gov (NCT02922101).

### Setting

The setting of our study is Dutch intensive care. In the Netherlands, virtually all ICUs are mixed medical-surgical closed-format units, i.e. units with the intensivist as the patient’s primary attending physician. Since 1996 Dutch ICUs can participate in the National Intensive Care Evaluation (NICE) quality registry [1]. Currently, all 32 teaching ICUs (of which 8 university ICUs) and 51 non-teaching ICUs in the Netherlands submit data to the registry and receive biannual written reports on indicating at least the severity of illness in their patient population, standardised mortality ratio, readmission rate and length of stay; each compared to the national average and the average of a group similar sized ICUs. Participants can also view these data, updated after each monthly data upload, on a website called NICE Online and perform subgroup analyses [41]. At the NICE coordination centre, dedicated data managers, software engineers and a coordinator are responsible for routine processing, storing, checking and reporting of these data. The NICE registry uses a framework for data quality assurance [42], including elements like periodical on-site data quality audits and automated data range and consistency checks. To participate in the current study, ICUs must submit an expanded data set needed to calculate the new indicators.

### Participants and data collection

All 83 ICUs that currently submit data to the NICE registry will be invited to participate in our study. They should be willing and able to submit the expanded data set monthly and allocate a QI team consisting of at least one intensivist and one nurse, of which one member is appointed “local champion” who is the key contact person for NICE researchers [43]. Managers and specialist nurses (e.g. a pain management coordinator) are suggested as additional members. The team is asked to spend at least 4 h per month on the intervention. The medical manager of the ICU must sign a consent form to formalise the organisation’s commitment. In the laboratory experiment (part 1), participants are the individual members of the QI teams, whereas in the RCT (part 2) participants are the ICUs.

We will use the existing data collection methods as currently applied by the NICE registry [44]. Data items needed to calculate the new quality indicators are aimed at not increasing registration burden and hence concern items already registered in ICUs’ electronic health record or patient data management systems. ICUs upload their data from their local database to the central NICE registry database through secure automatic data extractions.

### Intervention

**Fig. 2:**
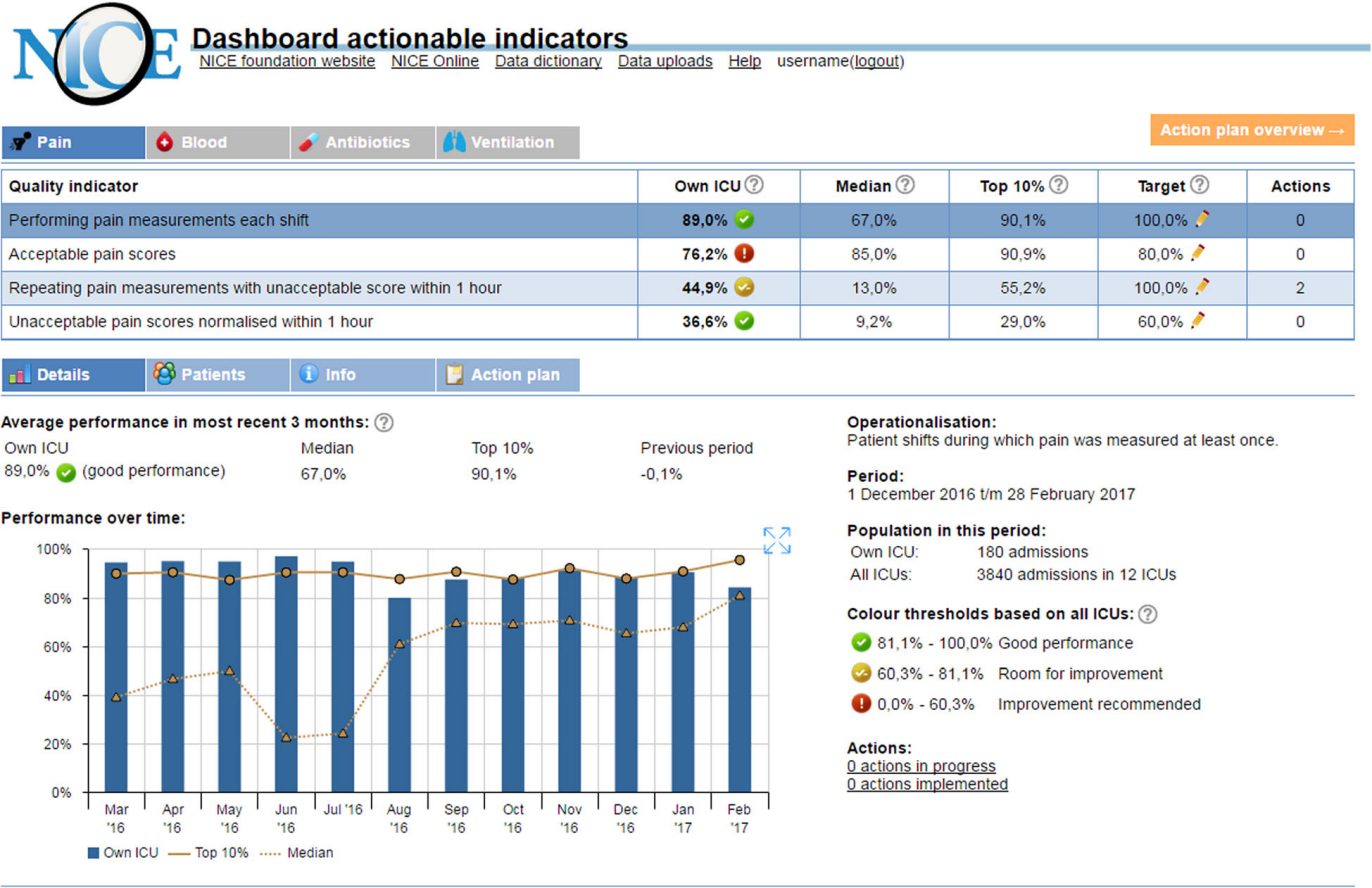
The NICE dashboard: detailed insight in clinical performance on quality indicators

All participating ICUs will receive access to an online quality dashboard (Fig. 2) that provides two key functionalities: (1) gaining detailed insight into clinical performance on quality indicators; and (2) developing and managing action plans. ICUs in the intervention group of the RCT additionally receive access to an integrated action implementation toolbox designed to further support the development and management of action plans. We designed the quality indicator set, toolbox and dashboard after careful review of the empirical and theoretical evidence in A&F literature and with continuous involvement by ICU clinicians. Additional file 1 summarises our intervention design by comparing it against Brehaut et al.’s [45] recent list of 15 A&F design suggestions.

#### Quality indicators and action implementation toolbox

Feedback will be provided on four pain management indicators that are listed in Table 1. We derived this indicator set using a modified RAND method. The method combines literature and guideline review with knowledge from ICU experts in an extensive rating and consensus procedure [46]. To address a potential lack of knowledge on how to improve on quality indicators, which was identified as an important barrier in the previous A&F study by our research group [21], a particular focus was placed on ensuring the actionability of the indicators during their development.

**Table 1:** Quality indicators used in this study; all fed back as proportions (nominator divided by denominator) with 100% being the maximum score

Quality indicator	Type	Unit of observation	Nominator	Denominator
Performing pain measurements each shift	Process	Patient shift	Patient shifts during which pain was measured at least once	All patient shifts
Acceptable pain scores	Outcome	Patient shift	Patient shifts during which pain was measured and no unacceptable pain scores were observed	Patient shifts during which pain was measured
Repeating pain measurements with unacceptable score within 1 h	Process	Patient shift	Patient shifts during which an unacceptable pain score was measured, and pain was re-measured within 1 h	Patient shifts during which an unacceptable pain score was measured
Unacceptable pain scores normalised within 1 h	Outcome	Patient shift	Patient shifts during which an unacceptable pain score was measured, and pain was re-measured within 1 h indicating that the pain score was normalised	Patient shifts during which an unacceptable pain score was measured

The action implementation toolbox comprises for each quality indicator (e.g. percentage of patients per shift whose pain is measured) a list of potential barriers in the care process (e.g. staff is unaware of the prevailing guidelines for measuring pain every shift), associated suggestions for actions to solve mentioned barriers (e.g. organise an educational training session), and supporting materials to facilitate implementation of the actions (e.g. a slide show presentation discussing the importance and relevance of measuring pain every shift). The toolbox’ complete content will be published elsewhere. The development of the toolbox took place in a parallel process to that of the quality indicators; similarly drawing from literature, guidelines and ICU clinicians’ expertise. In short, we used the Systems Engineering Initiative for Patient Safety (SEIPS) model [47] to identify the potential barriers in the care structures and processes that could lead to poor performance on each of the indicators. Next, for each barrier we determined a set of goal-oriented actions that may improve performance and collected supporting materials that could facilitate the implementation of those actions. The next paragraph explains how the toolbox is integrated in the dashboard.

#### The NICE dashboard

To gain insight into clinical performance, the NICE dashboard (Fig. 2) provides an overview of, for each quality indicator, the score achieved by the ICU, the median score of all participating ICUs, the average score achieved by the top 10% best performing ICUs [48] and a performance assessment represented by a “traffic light” coloured icon; all calculated over the most recent 3 months. Colour-indicated benchmark comparisons have been shown to help health professionals to set improvement intentions that are in line with the A&F recommendation [49]. Green icons (good performance) are assigned to performance scores above or slightly under the top 10% benchmark. If not green, yellow icons (room for improvement) are assigned to scores above or slightly under the median benchmark; red icons (improvement recommended) are assigned otherwise. The precise thresholds for assigning green or yellow lie *x* below the corresponding benchmarks, where *x* is the standard deviation (SD) of performance scores at ICU level with a ceiling limit of benchmark/10. For example, if the top 10% benchmark is 80% and the SD of performance scores is 20%, the threshold for assigning a green icon is 80 − 8% = 72%. This strategy was chosen to optimise the balance between providing sufficient “green” to prevent feedback rejection and providing enough “yellow” and “red” to encourage participants to undertake action. In particular, ICU clinicians involved in the dashboard design stated that they would consider performance scores that are just below the benchmark still “good performance”; receiving the recommendation to improve practice would seem unfair and could lead to recipients not accepting the feedback. From the dashboard overview, users can drill down to see detailed performance information, using trend charts displaying their own and peer performance over time, performance scores grouped by most relevant patient subgroups (e.g. only surgical patients; only patients during night shifts), and lists of individual patient numbers and whether or not the indicator was violated during a shift. The patient subgroup analyses and lists can be used to further investigate potential barriers in the care process and take corrective action where necessary [50], may increase trust in (quality of) the data [29], and have been previously identified as success factors in A&F [51]. Additional static information about the indicators are available, namely, their operationalisation, goal, relation to quality, definitions, inclusion and exclusion criteria, type (process or outcome) and unit of observation. Performance information is updated automatically each time an ICU submits new data.

To develop and manage structured action plans, users can navigate to the “action plan” tab. All ICUs can list their potential barriers in the care process and what actions they plan to undertake to improve. For each action, users can assign persons, set a deadline and record additional free-text details. ICUs in the intervention group of the RCT start out with an action plan that is prefilled with the toolbox’ list of potential barriers and suggested actions. The suggested actions are indicated by an icon of the NICE registry and include both a short description and detailed description justifying the action’s potential supported by relevant literature references. Some actions are accompanied by supporting materials to facilitate their implementation that can be directly downloaded through the dashboard.

### Procedure for part 1: laboratory experiment

**Fig. 3:**
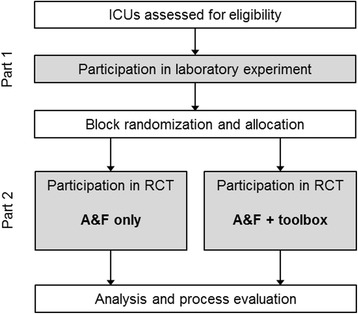
Study flow. *ICU* intensive care unit, *RCT* randomised controlled trial, *A&F* audit and feedback

The laboratory experiment takes place approximately 1 month before ICU teams receive their first feedback on the new quality indicators (Fig. 3). The experiment consists of two rounds which both take place using an adapted version of the NICE dashboard in which the action plan and toolbox are inaccessible. In the first round, the indicators and their static information (see Intervention) are presented, but measured performance information is withheld. Participants are asked to estimate for each indicator their own ICU’s performance score (perceived clinical performance; range 0–100%) and the average score in Dutch ICUs (perceived peer performance; range 0–100%), fill out the minimum performance score they would consider “good” performance (target; range 0–100%), and whether or not they would perform actions to improve upon the selected indicator (intention to improve practice; range yes/no). According to Control Theory [28], if participants make a negative self-assessment of their performance (i.e. perceived clinical performance < target) they will develop intentions to improve practice (i.e. intention = yes) and vice versa. If this hypothesis is violated (e.g. negative self-assessment but no intention to improve), participants are asked to explain their choice using a predefined list of reasons (Table 2) or in free text. The provided predefined reasons were developed guided by theoretical behaviour change frameworks [52, 53] and previous work [49, 54].

**Table 2:** Predefined reasons to be asked if hypotheses posed by Control Theory are violated

Hypothesis violation	Predefined reason
Negative self-assessment but no improvement intention	This indicator is not an important/relevant aspect of intensive care
	Actions will not improve our performance score on this indicator
	We lack the resources/time/knowledge to take action for this indicator
	Me or my colleagues cannot be motivated to take action for this indicator
	The benchmark (median/top 10%) is unrealistic/unfeasible (round 2 only)
	The measured performance score for our ICU is inaccurate (round 2 only)
Positive self-assessment but improvement intention	This indicator is an essential aspect of quality of intensive care
	It is easy to improve our performance score on this indicator
	Our performance is too low (round 2 only)

In the second round, participants are additionally exposed to all detailed performance information for the indicators including their own performance; the median and top 10% benchmarks; and past performance levels. Participants are asked the same as in round 1, but this time based on the feedback information at hand, their performance target (range 0–100%) and intention to improve practice (range yes/no). During this round, if improvement intentions do not match with the performance assessment presented in the dashboard (e.g. room for improvement [yellow icon] but no intention to improve), participants are again asked to explain their choice using the same list of predefined reasons as in round 1, extended with three reasons relating to feedback rejection (Table 2) or free text.

Finally, if there are discrepancies between improvement intentions in the first and second round (e.g. initially participants did not develop intention to improve on a specific indicator, but after receiving feedback they did), participants are asked what feedback elements caused this (measured performance score were higher/lower than expected; benchmarks were higher/lower than expected; there was a green/yellow/red icon; other [free text]).

### Procedure for part 2: cluster randomised controlled trial

ICUs enrol in the RCT after completing the laboratory experiment on the pain indicators (Fig. 3). Each ICU will receive one educational outreach visit aimed at explaining the dashboard functionalities, how data should be interpreted and how action plans can be developed. The visits will be undertaken by one of two NICE investigators (MRB or WG) with a non-medical but QI background. Further, brief semi-structured telephone calls will be held monthly with each ICU’s contact person to gain progress reports, motivate them to continue using the intervention and provide technical assistance if necessary. The structure of the visits and monthly calls will be the same for all ICUs, with the exception that the action implementation toolbox and its contents will not be mentioned to teams in the control arm. ICUs will participate in the RCT from 6 to 9 months.

#### Randomisation and allocation

We will randomly allocate participating ICUs to receive “A&F only” or “A&F with action implementation toolbox” in a 1:1 ratio. We will randomise ICUs using a block randomisation, with randomly permuted blocks of two or four each consisting of an equal number of interventions and controls. A researcher, who is otherwise unaffiliated with the study and blinded to the identity of the units, will perform the randomisation according to a computer-generated random schedule produced using an R script (R Foundation for Statistical Computing; Vienna, Austria) before the study starts. The size and the contents of the randomisation blocks will be concealed from the investigators enrolling the ICUs. Participants will not be told explicitly what aspect of the intervention is randomised; they will only be aware that there are two variations of providing A&F. Due to the character of the intervention, it is not possible to blind the investigators.

### Outcome measures

The primary outcome of the laboratory experiment (study part 1) is the proportion of improvement intentions set by participants that are consistent with A&F recommendations. We consider improvement intentions to be consistent with recommendations when participants intend to improve indicators with room for improvement (i.e. red or yellow) and when they do not intend to improve indicators without room for improvement (i.e. green). This measure was chosen because A&F should help recipients focus their efforts and allocate their resources on indicators for which improvement is recommended and not on indicators for which it is not. We will compare consistency within participants before and after receiving feedback. We will further report on the difference between perceived clinical performance (before receiving feedback) and measured performance; difference between performance targets set by participants (before receiving feedback) and the benchmarks determined by the A&F; whether set performance targets after receiving feedback tend to move to the median or top 10% benchmark; and reasons for not intending to improve on indicators despite a negative self-assessment (i.e. perceived clinical performance < target) and vice versa.

The primary outcome of the RCT (study part 2) is the proportion of patient shifts during which pain has been adequately managed, meaning that there was no unacceptable pain and that if there was acceptable pain, this was normalised within 1 h. This proportion reflects the composite performance of an ICU with respect to the individual pain management indicators, as detailed in Table 1. Our unit of observation is a patient shift; defined as a specific ICU patient during a specific shift (i.e. night, morning or day shift). Secondary outcome measures are the performance scores on the individual quality indicators underlying the composite score.

### Sample size

Sample size calculations for the RCT were based on pilot data of pain measurements from six ICUs (five teaching and one non-teaching) in 2014. Based on these data, the mean performance score for pain management is expected to be 76% with a standard deviation of 0.05%. We assumed a cluster size of 300 patient shifts per ICU per 3 months (3 months × 10 average number of patients × 10 average length of stay in shifts). The control arm, receiving only A&F, is expected to increase performance by a median of 4.3% (absolute improvement) based on the Cochrane review of previous A&F studies [18]. To have 80% power to find a difference in performance score for pain management of 10% using a two-sided unpaired *t* test with α = 0.05 would require a total of 24 ICUs to participate in the study.

We did not perform a sample size calculation for the laboratory experiment (study part 1) because this experiment will be conducted in the context of the RCT.

### Statistical analysis

Descriptive statistics will be calculated for all variables of interest. Categorical variables will be summarised using counts and percentages. We will use mixed-effects logistic regression analysis for the main analysis in both parts of the study.

To assess the influence of A&F on self-assessments and improvement intentions (laboratory experiment in part 1), we will use a binary “A&F received” covariate. To adjust for correlations between repeated observations within participants we will add a random intercept for “participating professional”. To adjust for clustering effects within ICUs and around quality indicators, we will add random intercepts for “ICU” and “quality indicator”.

To assess the effect of receiving the action implementation toolbox on pain management performance (RCT in part 2), we will use “patient shift” as unit of analysis. We will include the covariates time, study arm and the interaction term time × study arm, while adding random effects to adjust for clustering within ICUs, patients and shifts. If we suspect problems with the randomisation, we will perform tests of imbalance between groups in baseline variables that may influence the outcome, i.e. age, gender, length of stay and severity of disease at patient-level and hospital type and ICU-level. In case of an imbalance (above 5% level for statistical significance), we will conduct a sensitivity analysis adjusted by these variables to test the robustness of our methods [55].

### Process evaluation

We will perform a process evaluation using both quantitative and qualitative methods to gain insight in how the A&F intervention is implemented and used in clinical practice, and explore if and how implementation and use may affect the intervention’s impact [56].

For the quantitative part we will analyse usage logs of the dashboard to investigate the frequency with which the dashboard is used and by whom, and how this varies between ICUs and over time. We will also assess which areas of clinical performance (i.e. which indicators) users tend to focus on, and which feedback components (indicator details, patient subgroup analyses and patient lists) they typically access, and under which circumstances. Finally, we will study the contents of the action plans including the number of planned actions, whether those were typically selected from the action implementation toolbox or self-defined, and whether and when the actions are implemented. Analysing the usage logs of digital interventions allows us to study the intervention process quantitatively and unobtrusively [57].

For the qualitative part, we will collect data during the monthly telephone calls. Guided by quantitative summary reports drawn from the usage logs, we will investigate participants’ experiences with the dashboard, how they have implemented the dashboard into daily clinical practice (e.g. do they organise monthly team meetings to review the feedback; is the manager or intensivist involved; how much time do they invest in QI activities) and ICU- and individual-level barriers and facilitators that may affect the planning and implementation of actions or the intervention’s impact.

## Discussion

In order to learn how to design and deliver effective electronic A&F interventions, we need to understand the mechanisms of how A&F leads to improvements in clinical performance. Our study uses a theory-informed approach to investigate how A&F affects improvement intentions among ICU professionals (i.e. information–intention gap), and whether an action implementation toolbox with suggested actions and materials helps translating those intentions into action (i.e. intention–behaviour gap) in a two-armed RCT. The laboratory experiment, RCT and the comprehensive process evaluation are expected to provide insightful understanding of the mechanisms behind A&F.

### Strengths and limitations

The principal strength of our study is the extensive use of Control Theory [28] as a basis for our study objectives, design and evaluation. Although there is growing recognition that theory should play a central role in the design and evaluation of A&F interventions [27], explicit use of theory remains scarce [26, 58]. For example, the laboratory experiment (study part 1) will test the hypothesis that A&F improves self-assessments of clinical performance and hence improvement intentions; this hypothesis is typically assumed to be true in A&F studies but has not, to the best of our knowledge, been evaluated empirically. Also, while the large majority of quantitative evaluations A&F interventions solely report effects on clinical performance, our study will also explore the underlying mechanisms of A&F in an extensive process evaluation. Due to the electronic nature of our A&F dashboard, we are able to observe essential behavioural constructs such as intentions (planned actions), and behaviour change (completed actions), allowing us to quantify and study their relationships and potentially explain any outcome variation [57]. Further, because we will perform the qualitative part of our process evaluation guided by quantitative process reports, we may be able to make more effective and efficient use of our qualitative method by asking more focused questions.

We based the design of our intervention on theoretical and empirical evidence from A&F literature and carefully considered what feedback information to provide and how to present it [18, 23, 28, 30, 45, 59–62]. Although this increases the probability that the intervention as a whole will positively affect intensive care performance, we know from four decades of A&F trials that the effects are variable and often marginal [18]. Therefore, an RCT comparing the intervention to usual care would produce a limited amount of new knowledge [25]. In line with the international research agenda for A&F studies, our head-to-head comparison of different approaches to A&F delivery, with versus without action implementation toolbox, will contribute to speeding up the rate with which we identify the active ingredients of successful A&F interventions [27].

There are some limitations relating to the selection of participants in our study. Eligible ICUs are participating in the NICE registry, are capable of submitting the data items for the quality indicators and agree to allocate a QI team. These criteria may lead to the selection of a non-representative sample of ICUs, because eligible facilities are less likely to be understaffed and more likely to have information technology support to facilitate routine collection of NICE data. Therefore, the generalisability of our findings may be limited to ICUs that are motivated and equipped to systematically monitor and improve the quality of care they deliver. However, as information technology support are rapidly improving in most hospitals, we believe that this will be of less concern in the future. Finally, there is some evidence that non-teaching hospitals use pain assessment tools more often than teaching hospitals [12]. Even though applying a stratification method according to hospital type would equalise the distribution of hospital types over the two arms in the RCT (study part 2) and thus prevent confounding by this variable [63], we expect there will be insufficient participants to do so. Therefore, in case of an imbalance between arms at baseline, we will assess the robustness of our findings by means of a sensitivity analysis.

### Potential implications for practice and research

The results of this study will inform providers and managers in ICU or other clinical settings on how to use indicator-based performance feedback in conjunction with an action implementation toolbox to accelerate systematic local QI. Within the ICU context, this study will produce concrete and directly applicable knowledge with respect to what is or is not effective for improving pain management, and under which circumstances. If the study is successful, the dashboard and action implementation toolbox will be made available to all 83 ICUs in the Netherlands (100%) that currently participate in the NICE registry.

The results will also guide future research that aims to understand the mechanisms behind A&F and identify success factors of effective interventions. For example, if the laboratory experiment in study part 1 shows that ICU professionals’ intentions are not or rarely influenced by feedback, an implication is that more effort should be put in closing the information–intention gap before seeking to enhance any subsequent step in the A&F cycle. Alternatively, its feedback does effectively influence intentions, room for improving A&F interventions is more likely found in subsequent steps. Second, a positive result from the RCT in study part 2 will suggest that the addition of an action implementation toolbox effectively reduces the intention–behaviour gap. The process evaluation may then reveal how, e.g. because it helps ICU professionals overcome a knowledge, time, capacity or skill barrier to come up with or complete actions. An effective toolbox could take over the role of costly and labour-intensive co-interventions such as educational outreach visits while increasing intervention feasibility. However, a negative result from the RCT will suggest a need for a revision of the toolbox in terms of contents or usability, or alternative or more intensive approaches to facilitate ICU professionals to achieve their QI targets.

### Future research

Our research team is currently extending the set of quality indicators and the action implementation toolbox to cover blood transfusions, antibiotic use, and mechanical ventilation (e.g. [64]). ICUs that complete the RCT and submit all data items necessary to calculate the new indicators will all gain access to the toolbox and receive performance feedback on the new indicators in addition to the ones relating to pain management. Our future research will aim at validating our study results by means of an interrupted time series analysis and preceding laboratory experiment using these indicators.

## Additional file

Additional file 1: Summary of our intervention design by comparing it against Brehaut et al.’s [45] recent list of 15 A&F design suggestions. Note that the term “action” in Brehaut et al.’s table refers to the clinical feedback topic (i.e. indicators) whereas in this study we use “action” to indicate behaviour in response to receiving feedback. (DOCX 18 kb)

## Abbreviations

A&F: Audit and feedback
ICU: Intensive care unit
NICE: National Intensive Care Evaluation Foundation
QI: Quality improvement
RCT: Randomised controlled trial
SD: Standard deviation
SEIPS: Systems Engineering Initiative for Patient Safety
