# Supplementary material for: Electronic audit and feedback intervention with action implementation toolbox to improve pain management in intensive care: protocol for a laboratory experiment and cluster randomised trial
Source: Implement Sci. 2017 May 25;12:68. doi: 10.1186/s13012-017-0594-8 (PMC5445355; doi:10.1186/s13012-017-0594-8)
Supplement: Additional file 1: — Summary of our intervention design by comparing it against Brehaut et al.’s [45] recent list of 15 A&F design suggestions. Note that the term “action” in Brehaut et al.’s table refers to the clinical feedback topic (i.e. indicators) whereas in this study we use “action” to indicate behaviour in response to receiving feedback. (DOCX 18 kb) [file 13012_2017_594_MOESM1_ESM.docx]

**Supplemental file 1.** Summary of our intervention design by comparing it against Brehaut *et al.*’s [46] recent list of 15 A&F design suggestions. Note that the term ‘action’ in Brehaut *et al.*’s table refers to the clinical feedback topic (i.e. indicators) whereas in this study we use ‘action’ to indicate behaviour in response to receiving feedback.

| **A&F design recommendation** | **NICE dashboard intervention** |
| --- | --- |
| *Nature of the desired action* | |
| 1. Recommend actions that are consistent with established goals and priorities | We used a modified RAND method [35] to develop a set of indicators that are perceived by ICU clinicians to be relevant, feasible and actionable. |
| 1. Recommend actions that can improve and are under the recipient’s control | The indicators were selected based on relevance, feasibility, and actionability. Next, pilot data were collected from six ICUs to assess variation between ICUs and room for improvement. |
| 1. Recommend specific actions | For each indicator specific information is available including the nominator and denominator, goal (e.g. measure pain at least each shift), relation to quality, definitions, and inclusion and exclusion criteria. The action implementation toolbox further suggests concrete quality improvement actions. |
| *Nature of the data available for feedback* | |
| 1. Provide multiple instances of feedback | Feedback is provided through an online dashboard which is accessible 24/7 and by all team members. The team is asked to meet monthly to discuss new feedback. |
| 1. Provide feedback as soon as possible and at a frequency informed by the number of new patient cases | Feedback is automatically updated after each data upload by an ICU; which typically occurs monthly. The indicator scores and benchmark comparisons are based on the most recent 3 months of patient data to create a robust score that is steady over time. |
| 1. Provide individual rather than general data | Feedback is provided at the level of the ICU team rather than individual. ICU care is delivered by multidisciplinary teams and individual professional data are not collected. To increase feedback relevance we additionally provide patient-level feedback that can be used to look up additional information in ICUs’ local patient records. |
| 1. Choose comparators that reinforce desired behaviour change | Three external, data-driven targets are provided. Two are based on peer performance (median and top 10% benchmark) that provide achievable targets for both high and low performers; the third is past performance which can be used to assess progress. Finally participants set their own, internal targets guided by the information presented to increase target commitment. |
| *Feedback display* | |
| 1. Closely link the visual display and summary message | For each indicator the performance assessment represented by a “traffic light” coloured icon is displayed directly next to the measured performance score. |
| 1. Provide feedback in more than 1 way | Feedback is provided numerically (performance scores), graphically (coloured icons and charts) and textually (e.g. “improvement recommended”). |
| 1. Minimize extraneous cognitive load for feedback recipients | The intervention targets four indicators. The performance summary is always visible in the top half of the dashboard. The bottom half adopts a tab-based structure to separate information. Dashboard use is supported by both visual cues (icons; colours) and actionable messages. |
| *Delivering the feedback intervention* | |
| 1. Address barriers to feedback use | Medical manager of the ICU signs a consent form to formalise commitment. ICUs therefore consent to: allocating quality improvement teams with at least one intensivist and one nurse; the feedback is believed to be under control of these professionals. One team member who typically has QI in their portfolio is appointed local champion. The team is asked to spend at least four hours per month on the intervention, and to meet monthly to discuss new feedback and update the action plans. We motivate participants to continue using the intervention during monthly telephone calls. |
| 1. Provide short, actionable messages followed by optional detail | The dashboard first provides a general overview of current performance; after which details are available in a tab-structure (e.g. trend charts, thresholds of coloured icons, scores grouped by patient subgroups, patient lists). |
| 1. Address credibility of the information | Indicator calculations are made transparent by providing all definitions, nominators and denominators, and downloadable lists of individual patient numbers and whether or not the indicator was violated. |
| 1. Prevent defensive reactions to feedback | Multiple targets (median and top 10% benchmark) are provided to allow low performers to perceive achievable targets. Assignment of coloured icons depends on score and variation (see Intervention) so that performing just below the top 10% benchmark is still considered “good performance”. During the outreach visit and in the dashboard’s help function it is explained that colours are based on peer performance and designed to help participants quickly identify room for improvement; not to judge. |
| 1. Construct feedback through social interaction | During the outreach visit feedback is discussed within the ICU teams. Teams set their own performance targets in the dashboard guided by the performance information received. The team is asked to meet monthly to discuss new feedback and update the action plans. We hold monthly telephone calls with the ICU’s local champion to discuss progress and provide assistance if necessary. |
